# Participatory ethnobotany and conservation: a methodological case study conducted with *quilombola* communities in Brazil’s Atlantic Forest

**DOI:** 10.1186/s13002-019-0352-x

**Published:** 2020-01-13

**Authors:** Eliana Rodrigues, Fernando Cassas, Bruno Esteves Conde, Crenilda da Cruz, Eduardo Hortal Pereira Barretto, Ginacil dos Santos, Glyn Mara Figueira, Luiz Felipe Domingues Passero, Maria Alice dos Santos, Maria Angélica Silva Gomes, Priscila Matta, Priscila Yazbek, Ricardo José Francischetti Garcia, Silvestre Braga, Sonia Aragaki, Sumiko Honda, Thamara Sauini, Viviane S. da Fonseca-Kruel, Tamara Ticktin

**Affiliations:** 1 Center for Ethnobotanical and Ethnopharmacological Studies (CEE) - Department of Environmental Sciences, Universidade Federal de São Paulo (UNIFESP), Rua Professor Artur Riedel, 275, Jardim Eldorado, Diadema, SP 09972-270 Brazil; 2 Associação dos Remanescentes de Quilombo do Cambury, Ubatuba, SP Brazil; 3 Herbário Municipal (PMSP) – Secretaria Municipal do Verde e do Meio Ambiente, São Paulo, SP Brazil; 4 Associação da Comunidade dos Remanescentes de Quilombo da Fazenda, Ubatuba, SP Brazil; 5 Centro Pluridisciplinar de Pesquisas Químicas, Biológicas e Agrícolas [CPQBA] – UNICAMP, São Paulo, Brazil; 6 Institute of Biosciences, São Paulo State University (UNESP), São Vicente, SP Brazil; 7 Amerindian Studies Center, Universidade de São Paulo (CEstA-USP), São Paulo, SP Brazil; 8 Instituto de Botânica (IBt), São Paulo, SP Brazil; 9 Instituto de Pesquisas Jardim Botânico do Rio de Janeiro, Rio de Janeiro, Brazil; 10 Department of Botany, University of Hawai’i at Manoa, Honolulu, Hawaii USA

**Keywords:** Ethnobotany, Conservation, Participatory research, Participatory management, Atlantic forest

## Abstract

**Background:**

Although multiple studies advocate the advantages of participatory research approaches for ethnoscience, few provide solid contributions from case studies that involve residents in all of the project phases. We present a case study of a participatory approach whose aim is to register ethnobotanical knowledge on the use of plants in two *quilombola* communities (*maroon communities*), an important biodiversity hotspot in the Atlantic Forest, Southeast Brazil. Our aim is to provide tools that will empower decision-making related to sustainable use and management among residents.

**Methods:**

In phase I, the objectives and activities were defined in meetings with residents to carry out ethnobotanical surveys between two *quilombola* communities—the *Quilombo da Fazenda (QF)* and *Quilombo do Cambury (QC)*. In phase II, we offered community partners training courses on how to collect plants and ethnobotanical data. In coordination with the university team and using ethnobotanical methods, community partners interviewed specialists on plants and their uses. In phase III, using the participatory mapping method, residents indicated plot locations and collected plants to calculate the Conservation Priority Index for native species recorded in phase II.

**Results:**

In 178 days of fieldwork, two community partners from the *QF* and three from the *QC* selected 8 and 11 respondents who reported 175 and 195 plant species, respectively, corresponding to 9 ethnobotanical categories. Based on requests from the local community, booklets and videos with these data were collaboratively produced. A large percentage of species were found to be of great conservation priority—82.1% in the *QC* and 62.5% in the *QF*. *Virola bicuhyba*, *Cedrela fissilis*, *Plinia edulis*, and *Tabebuia cassinoides* are the species most at risk and will be the focus of phase IV, when a participatory management plan will be carried out. Additionally, we present both challenges and opportunities with the hope that others can learn from our successes and failures.

**Conclusions:**

Our experience shows that it is possible to train community members who wish to document their knowledge to support the process of ensuring that local knowledge is highly regarded, further ensuring its perpetuation. In this context, the project may be of great interest to development programs in promoting community-based management strategies for useful plants.

## Background

### Participatory ethnobotany and conservation

A combination of participatory and ethnobotanical tools related to data collection is being used in current ethnobotanical research, including workshops, focus groups, and field trips. In the current context, ethnobotanical research takes into account the relationship between people and plants, including cultural beliefs and practices associated with various forms of use (food, dyes, fibers, poisons, fertilizers, building materials, ornaments, oils, rituals, and others) and the conservation of the natural environment in accordance with the knowledge, practices, beliefs, and priorities of indigenous and local communities [1, 2].

Several authors have employed a participatory approach to ethnobotanical research [3–6], where the involvement and active participation of local residents has been instrumental in the decision-making process, the advancement of innovative solutions in co-management projects, and the production, use, and management of plant resources. In the Brazilian scenario, this fact has become increasingly relevant due to the negative political context of the past few decades regarding the conservation of both plant and cultural diversity [7]. Therefore, profound changes are truly needed to recognize and support the participation of local communities in activities directly related to conservation under Aichi Goal 18 as well as to achieve the goals of the Global Plant Conservation Strategy, since Brazil is one of the signatory countries of the Convention on Biological Diversity [7].

The transformation of the ethnobotanical approach into the broader context of ethnobiology is notorious. In the past, several studies focused on the documentation of plants and their uses (by researchers/ethnobiologists), with informants seen as the object of research. Recently, studies have been changing the approach, seeking to tell the history of biodiversity and not only cataloging it; the local community participates in collaborative research [8–10]

The International Society of Ethnobiology also emphasizes the importance of collaborative and participatory research. In its Code of Ethics, the issue of participatory research is valued, thus supporting traditional communities in conducting research within their own society; undertaking their own research, recordings, databases, and more for their own use; and proposing recommendations, as active participation and reciprocity are to the mutual benefit of all parties [11].

As discussed by Stepp [12], participatory research is becoming increasingly common in the social sciences, and ethnobiologists have contributed to this approach. Several authors describe and demonstrate ways of developing approaches in ethnobotanical research, which may help us to better understand local environmental knowledge [13–16]. These authors also highlight the importance of empowering local community members as consultants and collaborators in the research process and note that the participation of these community members also increases the chance of success in putting the findings of the research into practice. Similarly, Ticktin et al. [17] and Etkin and Ticktin [18] reinforce the need for community members to actively participate in all phases of the research process, from study design to data interpretation. In recent decades, the importance of biocultural conservation, which employs participatory approaches, has received ever-increasing recognition, especially after the incorporation of the guidelines established in the Nagoya Protocol, which emphasizes that co-research (that actively involves local researchers in all stages of research and publication) that is carried out with the participation of the local communities and where the actors are not the object of study but take an active part in both researching and returning the information to the original owners, is more effective [16, 19, 20].

Common tools used in participatory ethnobotanical research include participatory mapping, considering community participation, and contributing to more adaptive landscape planning and conservation of forest and livelihood resources [4, 21]. Other authors have employed methods of participatory photography to portray changes in the local environment as well as adaptations to climate change through participant photography and accompanying explanations made by the local people [15, 16, 22]. However, there are few published studies in which research has involved local residents from the study design and data recording through the analysis. Hitziger et al. [23] provide one of the few examples of this, focusing on the development of a large-scale cooperative research project in ethnopharmacology. Their project was conducted in Guatemala among the Kaqchikel (highland) and Q’eqchi’ (lowland) Mayans. As ethnobotanical research shifts from the documentation of plant knowledge and uses to other areas [24], including applied research on resource management, these approaches are critical, and it is important to learn from both successes and challenges [17].

The Brazilian Atlantic Forest, a biodiversity hotspot [25] and one of the most endangered biomes in Brazil [26], originally extended for 3,300 km along the coast [27], where it has historically housed much of Brazil's human population. However, there has been heavy fragmentation caused by agriculture, livestock, firewood and urban sprawl [28], with only 8% of the original forest cover remaining [25]. There are still traditional communities that know about the use of plants such as the *quilombolas* (*maroon communities*). In this biome, the development of research that seeks the sustainable use of plant resources is a key priority for both human livelihood and the maintenance of forest biodiversity [29, 30].

This approach to participatory ethnobotany has been implemented with the support of local communities, including those who have resided in these areas, even before the creation of the integral protection area. This type of protected area has been implemented in Brazil in some areas since 2000 (Law No. 9,985 - National System of Conservation Units in Brazil/SNUC). The implementation of these protected areas causes various levels of conflict between the management of these areas and traditional populations that often have inhabited these localities since before the creation of the park, that is, there is conflict between conservation of the natural environment and the protection of the cultural rights of these human groups. To this end, the Brazilian government has adopted models of shared management between the federal government and traditional groups to reduce conflict. Thus, the present study aims to support actions and generate integrated knowledge based on sustainable management plans for better use of local plant resources.

We present a case study of a participatory approach whose aim is to record ethnobotanical knowledge on the use of plants in two *quilombola* communities (*maroon communities*) in an important biodiversity hotspot, the Atlantic Forest, in south-eastern Brazil, seeking tools to empower decision-making related to sustainable use and management among residents. First, we describe our research process and our project outcomes to date, and then we discuss some of the challenges involved. By specifically sharing the process here, as opposed to the outcomes produced by the project, we hope that others can learn from both our successes and our failures.

### Location and context

The Atlantic Forest is one of the five main biomes in Brazil. It is considered one of the richest areas of fauna and flora in the world, with approximately 20,000 species of plants, of which approximately 8,000 are endemic. Part of the Atlantic Forest was recognized by UNESCO as a biosphere reserve in the early 1990s. It is vital to find alternatives for maintaining local communities, such as those of artisanal fishermen, *quilombolas*, and small farmers, that have lived for over 150 years in the Atlantic forest biome and have used the natural resources available for generations. Thus, one of the challenges is to perpetuate local knowledge and simultaneously promote income generation for the conservation of this biome.

However, some situations have led to a reduced conservation status of the Atlantic Forest, especially the alteration of the forest code in 2012 (due to the expansion of agribusiness policies in Brazil), which has resulted in the increased social and environmental vulnerability of local communities that depend on these environments and thus compromise the survival of traditional communities [31].

**Fig. 1:**
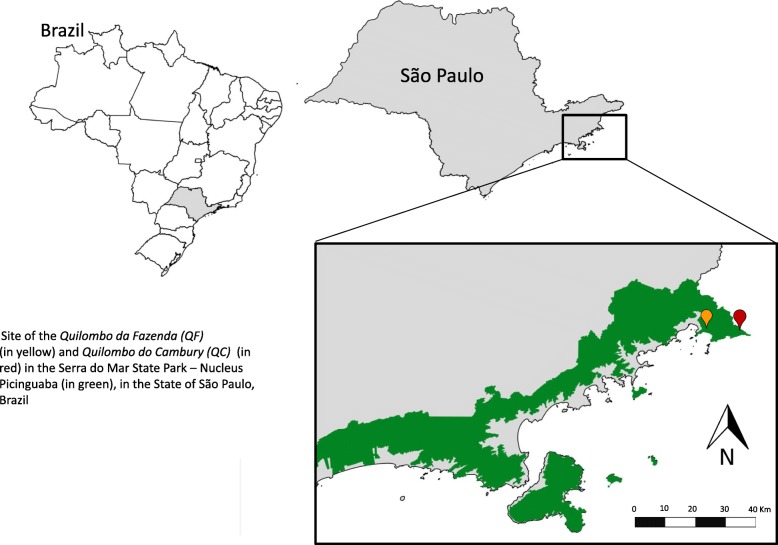
Site of the *Quilombo da Fazenda* (*QF* (*in yellow*) and *Quilombo do Cambury* (*QC*) (in red) in the Serra do Mar State Park – Nucleus Picinguaba (in green), in the State of São Paulo, Brazil

Our research focused on two *quilombola* communities (certified by the Fundação Cultural Palmares since 2005): the *Quilombo da Fazenda (QF)*, which dates back to the end of the nineteenth century and today is composed of some 40 families (170 people) and overlaps with a protected area known as the Picinguaba Nucleus of the Serra do Mar State Park; and the *Quilombo do Cambury (QC)*, which dates back over 150 years and today has approximately 50 families (230 people). The latter is located in the same park and Serra da Bocaina National Park, both located in Ubatuba Municipality, São Paulo State, Brazil, in the Atlantic Forest (Fig. 1).

*Quilombolas* are “remnants of *quilombola* communities” and are of African descent; we adopted the concept as it extends to *maroon communities*, including territory and identity linked to resistance, which are crucial elements in determining these remaining *maroon* groupings [32]. However, the term *quilombolas* is related to *quilombo* lands, which are ethnic-racial territories with collective occupation based on ancestry, kinship and cultural tradition, that express resistance to different forms of domination. Land regularization here is still guaranteed by the Federal Constitution of 1988 [33, 34]. *Quilombolas* were recognized by the National Policy for the Sustainable Development of Traditional Peoples and Communities, established by Decree 6040/2007. Some of them fled the farms where they were exploited and organized in communities, known as *quilombos.* They survived based on agriculture and the use of forest resources. This term is based on political, legal, sociological, anthropological and economic history, since it is associated with the period of colonization and slavery. In the context of history, it has been used in the legal arena to disqualify litigation by self-identified groups as *quilombolas* [35]. Over time, these communities have developed detailed local ecological knowledge and belief systems on the relationships of living beings [36]. The *QF* and *QC* communities are located in protected areas where there are multiple restrictions on land use, including the prohibition of resource extraction without authorization of the protected area manager. Currently, to construct houses, boats and handicraft, only dead or fallen trees may be used, pending permission of the parks. Inhabitants of the *quilombos* have pointed out the difficulty of rebuilding their homes, which degrade over time, and of using wood in general, due to park bureaucracy, as the park must grant them a permit to use wood.

This story is repeated in several areas of Brazil. The implementation of the National System of Conservation Units (SNUC) [37], specifically the implementation of protected areas, such as integral protection areas (ex. national parks), where only the indirect use of natural resources is allowed, created a process that is negligent regarding the extensive and diverse cultural heritage existing in the still representative strongholds, such as in the remnants of the Atlantic Forest biome, and neglects potential collaboration with local communities for the conservation and management of biological diversity [38–40]. This scenario has led to the abandonment of land cultivated by traditional farmers who migrate to periurban areas, leading to disorderly urban growth and the consequent irregular occupation and increase in underemployment and crime rates [41].

The largest environmental threat faced by the communities is pressure for the non-recognition of these areas as *quilombola* lands by the government, thereby allowing for the transformation of these areas into private property without considering conservation of the environment. If these areas are privatized, traditional populations will probably lose their land rights, and without land, they will not be able to carry out their traditional practices, as has happened along other areas of the coast of Brazil. The fishermen (*caiçaras*) from São Sebastião Municipality*,* São Paulo, for example, had to sell their houses since they could no longer pay the taxes demanded by the government. Tourists bought their homes, and the fishermen were turned homeless in their own land [42–44].

## Methods

### Ethics

It took 12 months to obtain all the proper permits, and data collection was only begun in 2016. Nevertheless, visits were conducted in the field to maintain a relationship with the community. Nonetheless, some community members who had been willing to participate in research in 2015 were not able to participate in 2016, either because they had since had a child or because they had obtained jobs outside the community.

### Team and community reach

This project involved the collaboration of members of the two communities (the *QF* and *QC*) and a team from several universities (national and international) and the Botanical Gardens in Brazil. On the part of the university, this included thirteen university researchers with expertise in ethnobotany, botany, taxonomy, anthropology, phytosociology, ecology, pathophysiology and agronomy, including six undergraduate and graduate students. On the part of the communities, this included five community partners, who actively participated in all phases of the project (from genesis and data analysis to publication); 19 interviewees took part in the project directly, and some 40 did so indirectly during filming, workshops, assemblies, and other activities developed with the communities.

### Project Phases

#### Phase I (2015)—genesis of the project

The first phase started in March 2015, with a workshop organized by the managers of the Picinguaba Nucleus of theSerra do Mar State Park, *Ubatuba*, SP State, Brazil, where different groups participated. During this event, the need for managers to encourage the development of projects related to local biodiversity and social and cultural aspects, including economic alternatives for the residents, was clear. Therefore, from April to June 2015, five meetings were held in the two *quilombola* communities (the *QF* and *QC*); these meetings involved community members and the university research team and aimed to develop the collaborative research with goals that would be of common interest.

During these meetings, members of the *quilombos* expressed the desire to inventory and record their knowledge in the form of a booklet and a short video about their culture because they feared the loss of this knowledge, since several older members had died or were very old. In addition, members of the community have acknowledged that this knowledge is threatened by its traditionally oral transmission due to the lack of interest of young community members in learning about practices/uses. It is noteworthy that these records should aid in the perpetuation of local culture and increase self-esteem in the communities because the *quilombolas* communities are under strong pressure due to discrimination in Brazil.

In the *QF*, two other requests were highlighted by the residents: they expressed interest in creating educational and touristic trails in a participatory way and stressed the importance of medicinal and/or cosmetic plants for their commercial value, aiming at local income generation and the production of management plans, within the perspective of the local community. The residents are interested in integrating their knowledge because the lands that the *quilombolas* occupy became a state park in the 1970s, thus affecting their economic activities and subsistence. Residents must leave the community to seek employment in the city of *Ubatuba* (50 km from the community) due to a lack of employment alternatives in the *quilombo* and the lack of schools in the community.

In this sense, the universities and research institutes, such as Herbaria, have been working to collaboratively build bases for the development of sustainable management plans for species of socioeconomic interest to the *quilombolas* through this project. In the near future, the establishment of terms of conduct adjustments for plant extraction in areas of the park, such as the *taboa* (*Typha domingensis* Pers., Typhaceae), one of the plants most indicated by the *QF* due to the importance of its use in local handicrafts for decades, is expected. Community members have methods and knowledge passed on for generations about when, where and how to collect the *taboa* to ensure that its use is sustainable. It is noteworthy that in Brazil, native, exotic or even cosmopolitan species that grow in protected areas require a management plan for their use [45, 46]. In the *QC*, no support has been requested in this regard, but throughout the meetings, interest has been shown in developing management plans for species that they deem interesting.

#### Phase II (2016–2018)—participatory ethnobotany

The following actions were selected to meet the goals identified by community members: (1) the production of a booklet and a short film; (2) the creation of a sustainable management plan of economically important plants; and (3) the construction of a tourist trail focused on medicinal and cosmetic plants. The university team suggested starting the study with ethnobotanical surveys to record knowledge, since that knowledge is the basis of all the other goals. University researchers and the Herbaria botanists team offered training on plant collection and presented anthropological methods to the community partners. To facilitate interactions among the university and community teams, the university team rented a house in one of the *quilombos.*

Community partners selected some of the residents for the interviews based on the criteria of “being an expert at least in one of the following categories: construction, medicine, food, handicraft, fuel, or ink, among others.” The community partners themselves collected ethnobotanical information through unstructured interviewing techniques [47], seeking information on socio-cultural aspects, detailing ethnobotanical data (common plant name, part used, type of use, method of preparation, link between plant collection and moon phase, possible gender-related collection restriction and collection instructions) and especially medicinal plants (prescribed plant parts, amount and method of preparation, route of administration, time of use and possible contraindications). Complementary activities in the training process, such as participant observations (POs) recording their daily perceptions in a field book, were performed by the university staff [47, 48]. Community partners collected plant samples and testimonies of ethnobotanical knowledge recorded from the interviews.

However, the local community preferred not to keep these samples in an herbarium and/or collection in their local communities. They preferred to have only photographic records, descriptions and plant information integrating local and scientific knowledge in leaflets and audiovisual material. With this, the collected plant samples were processed, planted, taken by the university researchers, and deposited at the São Paulo Municipal Herbarium (PMSP) and the Forest Institute (SPSF). Regardless of their interest in the collection and identification process, members of the local community, who are monitors at the Serra do Mar State Park, have been using their botanical knowledge in their work, which is encouraged by the university staff (who have been sharing knowledge and/or books on native Brazilian plants).

Since June 2015, monthly meetings of the university team and community members in the two communities, as well as eight workshops called exchanges of know-how, have involved the constant re-planning and re-evaluation of our project processes and outcomes every semester in both *quilombos*. We can illustrate the importance of this with an example: at the beginning of this project, local communities did not discuss the need for community partners to receive financial compensation for their work, but as the project progressed, they realized that budget forecasting was needed for this hourly compensation. Another example was the nature of the booklet: although the communities requested a booklet from the outset, the nature of the booklet was not decided upon. Only after data collection did the communities develop their ideas of what to focus on. For example, the *QC* inhabitants selected the top 10 most cited plants by category of use to be recorded in the booklet. In the *QF*, they had already decided to produce two leaflets: one containing all registered medicinal plants and another for plants in the other categories of use. Thus, the participatory approach involved the exchange of knowledge in both directions. Local knowledge was being registered by the community itself, which always expressed the need to include scientific knowledge in the outcomes produced by the project. For example, the communities asked university staff to include information on the toxicity of medicinal plants from the scientific literature in the leaflets. To verify, compare, and analyze the data, workshops were held with university staff and community partners. In addition, during these collaborative workshops, the team decided how to organize the data and prepared the brochures and videos.

#### Phase III (2017–2018)—conservation priority index for the native species

The native plants collected in phase II were evaluated in both *quilombos* using the Conservation Priority Index [49], which integrates the ethnobotanical, ecological, phenological, and bibliographic data of each plant’s conservation status. Ecological data collection involved the participatory mapping of harvest areas [36], followed by botanical surveys carried out by both university and community partners. This information helped in the selection of species for the management study, as did considering the economic aspects of these plants for the community. Specifically, while the *QF* community members had already selected one of their desired species, *taboa* (*Typha domingensis* Pers., Typhaceae), the others were selected by identifying those that are of the greatest interest to the community in generating income (handicrafts or cosmetics) and also have a high availability and conservation status. The residents were not trained to calculate this index, since none of them demonstrated interest in it.

#### Phase IV (2019–2021)—participatory management plans

During this period, the team is collaboratively conducting ecological and economic analyses to develop sustainable management plans for the four selected species.

## Results

The scheme below provides information on the various phases of the project as well as the respective outcomes produced by the project. Also, a video has been produced reporting the four phases of the project. It is available at Biota Program Youtube Channel: https://www.youtube.com/watch?v=-Q2hk1eHEj0.

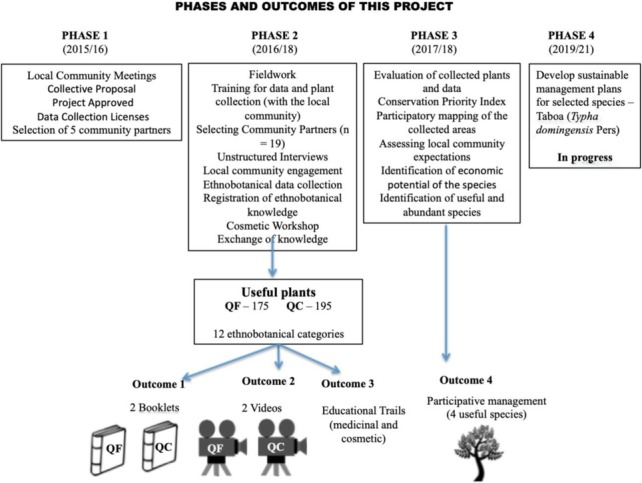



### Phase I

This phase and phase IV did not produce results, the former because it refers to the preparation of the participatory research and the latter because it is in progress at the moment and should be completed in 2021.

### Phase II

As of June 2018, in 178 days of fieldwork (see photos - bit.do/cee4, bit.do/cee5 and bit.do/cee6), 19 community members were interviewed by five community partners. The 8 interviewees from the *QF* generated a list of 175 plants; in the *QC* 195, plants were cited by the 11 interviewees. These plants were grouped into 9 categories of use. In both *quilombos*, the most numerous uses were medicines, foods/spices, and construction (Table 1). Only 14.6% of the species were reported by both *quilombos.* The categories of medicines and food/spices stand out for having the most species in common across the two communities, with 48.7% and 28%, respectively. Although both human groups are *quilombolas* and residents of the Atlantic Forest, one of the *quilombos* lives in the backlands and the other at the shore, that is, they live in different environments, from the point of view of plant physiognomy. Moreover, the history of the formation of each of these human groups is particularly influenced by different migratory flows. For example, while the *QC* has had Italian influences, the *QF* has not.

**Table 1:** Number and percentage of plant species belonging to the 9 ethnobotanical categories indicated by the 11 interviewees of the *Quilombo do Cambury* (*QC*) and eight of the *Quilombo da Fazenda (QF)*, and the coincident ones. The species indicated in each *quilombo* total 195 (*QC*) and 175 (*QF*). As the same species may belong to more than one ethnobotanical category, they total 318 and 244 species, respectively

Ethnobotanical categories	No. species cited in *QC*	No. species cited in *QF*	Total species cited in *quilombos*	No. and (%) species coincident in both *quilombos*
1. medicines	82	92	174	40 (48.7%)
2. food/spices	72	59	131	23 (28%)
3. construction	44	32	76	10 (12%)
4. shipbuilding	42	04	46	2 (2%)
5. handicraft	33	11	44	3 (3.7%)
6. technology	24	23	45	2 (2%)
7. combustion	18	06	24	2 (2%)
8. ritualistic	01	17	18	0 (0.0%)
9. ornamental	02	00	02	0 (0.0%)
Total	318	244	560	82 (14.6%)

The residents, along with the university team, devised a trail of medicinal plants. The construction of a tourist trail focused on medicinal and cosmetic plants will allow residents of the *QF* to receive tourists to generate local income. The residents did not want the species to be marked with nameplates, explaining that the identification of plants with plaques could threaten the species, since tourists could deplete populations by indiscriminately collecting as much as they needed and without criteria. The community members intend to talk about the conservation aspects of the plants with the tourists while walking along the trail. Some observations made by the residents demonstrated their concern for conservation. According to some residents, trees such as the *jatobá* (*Hymenaea* cf. *altissima* Ducke), *cambucá* (*Plinia edulis* (Vell.) Sobral), *canudo-de-pito* (*Mabea piriri* Aubl.) and *timbuíba* (Fabaceae) have been flowering early in recent years. For example, *canudo-de-pito* used to flower in January, but in recent years, it has flowered in October. The community members argue that precipitation has varied greatly over the years, thus interfering not only with the flowering of some plants but also with the river flood regime and soil quality. This is just one example of the residents’ concern for conserving their environment. In addition, a store selling forest products (candles, ointments, perfumes, soaps) made with the native plants present on the trails is being developed so that tourists can buy forest products that are not found in common outlets. To do this, the university team offered workshops to the community on making soaps, perfumes, candles, ointments, and repellents. The goal of this was to provide the *quilombolas* with another source of income from the sale of these products.

The booklets (*QF*: https://issuu.com/pbyazbek/docs/livro_-_quilombo and *QC*: https://issuu.com/thasauini/docs/livreto_cambury_thamara_final_13.06) and videos [50, 51] act to conserve biocultural heritage. They allow the recording of local knowledge and practices and provide a record for the communities’ descendants to access the culture of their ancestors, strengthening the maintenance of their knowledge, promoting autonomy over their intellectual property and facilitating decision making on which plants the community can use for economic purposes. In particular, the video is seen by the community as a record, not only for future generations but also for wider Brazilian society and beyond, of their cohesion as a culture and their resilience in living in an area under great environmental pressure. Since the video includes knowledge and uses of a wide range of members of the community—women and men, old and young—the final product is very inclusive. For example, in the words of one community member, “It [the project] was very beautiful because everyone in the community participated in some way.” Another member explained in a tone of pride, satisfaction and relief that “the knowledge that was left is no longer lost, is not lost anymore because it is filmed, it is written in the booklet and all this is already available on the internet too.” Another community member said that he was happy because his children and grandchildren and others to come will know that he is one of the few people in the community who still knows about plants for shipbuilding, “and they can use that knowledge when I am no longer here (after my death).”

### Phase III

Of the 78 native species analyzed in the *QC*, 64 (82.1%) were classified as having a high conservation priority (category 1), while in the *QF*, 62.5% fall into this category (40 of the 64 species). Taking into consideration those species in category 1, plus the list of endangered species for the State of São Paulo, *bicuíba* - *Virola bicuhyba* (Schott) Warb, Myristicaceae; *cedro-rosa* - *Cedrela fissilis* Vell., Meliaceae and *cambucá* - *Plinia edulis* (Vell.) Sobral, Myrtaceae held the highest relevance for the *QC,* while *caxeta* - *Tabebuia cassinoides* (Lam.) DC., Bignoniaceae and *cedro-rosa* - *Cedrela fissilis* Vell., Meliaceae were important for the *QF* (Table 2). The first two species are endemic to the Atlantic Forest and are threatened by extinction according to the CNCFlora (Center for Plant Conservation) [52]. These four species are potential candidates for a sustainable management plan so that they can be used by *quilombolas* and their local existence can be guaranteed.

**Table 2:** Ethnobotanical uses of the four species most at risk - according to the Conservation Priority Index, integrated to ecological, phenological and bibliographic data of each plant’s conservation status - for the native species recorded in phase II of this study

Vernacular name	Species (voucher)	Ethnobotanical use
*bicuíba*	*Virola bicuhyba* (Schott ex Spreng.) Warb - MA113	Shipbuilding, construction, combustion.
*cedro-rosa*	*Cedrela fissilis* Vell.- SB34	Shipbuilding, construction.
*cambucá*	*Plinia edulis* (Vell.) Sobral - MA100	Food and combustion.
*caxeta*	*Tabebuia cassinoides* (Lam.) DC. - GDS41	Shipbuilding.

### Contributions of the Project

This is a pioneering project developed collaboratively by the local residents and the university to support sustainable economic alternatives for communities while also conserving biocultural diversity. The interaction between universities and other research institutes and the knowledge of traditional communities can promote conservation and local development, mainly in regions under great environmental pressure. This participatory approach can serve as a model for other groups in Brazil and elsewhere, especially when dealing with issues related to the loss of indigenous and local knowledge.

#### Strengths and weaknesses

The strengths of this study are its multidisciplinary approach, combining different areas related to the use of plants, health, the conservation of both culture and diversity of local plants (through the elaboration of participatory management plans), and local income generation, in addition to engaging local and scientific knowledge on a fair basis. Weaknesses include a possible bias for the involvement of women, the unemployed and elderly persons, who are likely to spend more time at home than others. As we depended on the availability and interest of people to collaborate and perform the research, our sample could not be strictly random. Additionally, because few people have reliable records of birth and death so this information is often not available, we count on the interest of certain collaborators for this information. Our results are not representative of all *quilombolas* communities in Brazil because in addition to Brazil being of continental dimensions, there is a heterogeneity of areas occupied by different *quilombolas* in the country. Outside the main nearby city, *Ubatuba* (SP), health care facilities are more limited and poverty rates are higher. Thus, there are a lack of participative studies and studies that analyze and compare the knowledge of the *quilombola* communities on Brazilian biodiversity.

## Discussion

### Challenges

While our project has had many successes, it has also had challenges. One challenge was the length of time the permission process took and the difficulty that it posed for developing the participatory approach. It took 12 months to obtain the four required authorizations/registrations from the appropriate authorities to start the project since each application process had a particular time frame, yet one authorization was often dependent on another. This meant that the community had to wait a long time to start the project, which, to some extent, brought uncertainty and discouragement to all those involved (both the researchers and members of the community). Some of the residents gave up on the project during that time, although the university team made field visits throughout this period and were therefore available for communication. This situation may also be the case when, after learning about a community’s interests, researchers need to submit grants for funding. In this case, it can take many months.

Second, while our project involved strong participation from many community members, many others did not participate. This was due to a number of different reasons. One reason is that, since community members are not allowed to use the land, they all have to work outside the community, depending on public transport to travel long distances to work every day to make a living, which leaves them with little free time for other activities, such as those related to this project. This lack of time is common in most places, including those where people need to carry out subsistence activities throughout the day. The lack of local employment has opened up space for drug use in the community, with disruption in relationships between young people and their parents and grandparents. Community participation was also inhibited by internal community relationships, such as historical conflicts between families or financial issues within their associations. These conflicts can reduce or inhibit community participation in meetings and collective activities, with some community members refusing to participate in activities that include certain other members of the community. Third, while collaborative teams made up of individuals from the university and the community can be highly effective, a collaborative process can also be slow, especially when team members have limited time (in this case, community partners had many other responsibilities to attend to) and when the project outcomes are dependent on the participation of all members. This limitation can also pose a challenge for grant reporting and/or securing future funds.

Finally, the context of the communities living within a protected area made some of the information difficult to share broadly. The relationship and communication with the managers of the parks where the *quilombos* are tend to be bureaucratic and restrictive. A practical example of a difficult situation arising from this problematic relationship is that it is difficult to record community knowledge on the use of wood for canoe construction when the use of wood from the environment is prohibited.

Lessons learned from, and guidelines for, participatory approaches have been reported by other studies [6, 21, 23, 53]. Below, we highlight some important points to consider in participatory ethnobotanical research that emerged in our project.

1. Plan community activities according to real needs and beliefs and recognize that these change. If a project does not address the needs of local residents, its outcomes will likely be ignored. We were able to do this in our project through an adaptive process, as needs and interests changed over time. Shrestha and Medley [21] demonstrated the importance of tools such as participatory mapping in elucidating the needs that are important to the community (for example, participants mapped places of symbolic importance because they are associated with spiritual beings, stories, myths, and rituals that supported their protection). If this information had been ignored, the participatory project would probably have failed because local beliefs about sacred spaces would not have been considered, as they should primarily be respected and should not be used in a management plan, for example.
2. Involve residents in all stages of the research process. Our process involved engaging residents throughout the project, from conception to the dissemination of the results. Similarly, Hitziger et al*.* [23] developed a participatory ethnobotanical project. In their project, the Councils of Elders (local people) in Guatemala suggested documenting traditional phyto-therapeutic knowledge to strengthen the identity of Mayan medicine, to build societal awareness, and to preserve knowledge for future generations. The council members took part in the project planning since the project’s inception, with objectives, research design, and sampling strategy jointly designed among local people and investigators. Additionally, Paniagua-Zambrana et al. [15] involved people in indigenous communities in Chácobo, Bolivia, in documenting local knowledge. By exploring the effects of interviewer identity and knowledge on the elicited plant species and uses in Chácobo, Bolivia, these authors suggested that the training of indigenous interviewers and plant collectors should be a serious consideration when conducting any studies involving the documentation of traditional knowledge. The authors also suggest that the combination of indigenous interviewers and a very large set of participants is an excellent strategy to elucidate a maximum amount of information in ethno-botanical studies. In these three projects, this process deepened the degree of responsibility and the sense of belonging of the local residents towards the research phases as well as the results and outcomes of the projects. However, in their review of participatory projects in natural resource management, Johnson et al. [6] report that researchers tend to maintain control in the initial decision on how the target group and clientele are defined and in the diffusion phase when decisions on dissemination on a wider scale are made. Thus, these issues need special consideration when designing participatory projects. In our project, some difficulties arose during the completion of the sheets; one of the community partners is illiterate, and since she could not write, the university team helped her in recording the data. There was a lack of interest on the part of community partners in the use of electronic equipment. Thus, the researcher and the university team responsible gave the necessary support, committing themselves to always sharing photos, edited videos and the recorded data in the computer. This helped the community partners to better understand how the data are processed after the field interview and to visualize the importance of each step until the finalization of the project in the format of the primer that was created. Not everyone had ability or interest in filming, but photos were the medium of record that appealed to most of the community members.
3. Discuss compensation for community partners from the project initiation. Researchers are usually compensated for their time in participatory projects, and community partners must also be, in whichever ways they decide are the most appropriate (e.g., financial or other). This issue should be discussed from the start. In our experience, the community partners did not initially mention financial compensation, but then one of the *quilombolas* decided it was important once the project was initiated. We therefore adapted our process to include such compensation for both communities.
4. Be aware of bias in selecting local residents to participate in the project. As mentioned above, the community partners selected some of the residents for the interviews based on the criteria of “being expert in at least one of the following categories: construction, medicine, food, handicraft, fuel, or ink, among others.” As Johnson et al. [6] argue, this process of selecting residents in participatory research determines what information is collected; therefore, in the outcome analysis process, care should be taken to avoid generalizations and/or extrapolations. The authors also point out that the ability of a community to identify appropriate participants may also depend on its ability to make collective decisions. If it is perceived that participation in a project will bring material or social benefits, community leaders may nominate their allies. Based on an inventory of 59 self-described participatory R&D projects in an area of natural resource management, Johnson et al. [6] observed that only 2% of the projects selected participants exclusively on the basis of equity criteria. These results suggest that if marginalized groups are less likely to be identified by these types of selection methods, then these groups will be excluded from most participatory natural resource management projects. Hitziger et al. [23] also point out that very few ethnobotanical studies have so far critically reflected upon the strategies of informant selection as part of the research process. Their informant selection was a two-step process designed to access “emically eminent phyto-therapeutic specialists.” In the first step, the university researchers selected the Kaqchikel (highlands) and the Q’eqchi’ (lowlands) groups to represent important floristic zones due to preliminary data on the relevance of phytotherapy in their respective medical traditions and to practical reasons such as reliability, internal organization, legitimacy and rapport with healers. In the second step, the respective councils chose healers with locally reputed specialist knowledge in phytotherapy. The strategy thus took advantage of local knowledge and the perceptions of healers’ skills, experience, and reputations.
5. Make sure the activities strengthen the ability of communities in a move forward for future planning. This is one of the greatest challenges in participatory research. Johnson et al. [6] created an inventory recording data on 59 participatory projects. They discussed these data based on the five basic principles for good practices in participatory research for natural resource management identified by Vernooy and McDougall [53]. Considering the fourth principle, “The research contributes to concerted planning for the future and social change,” Johnson et al*.* [6] conclude that the projects they reviewed were well oriented and organized internally, building on solid methodologies for how farmers and researchers can work together in the context of specific research projects. However, they identified that a clearer idea of how the outcomes and capacities developed by the projects were expected to contribute to the broader on-going development process was missing. In our project, it is difficult to say whether there will be initiatives begun by the communities from the joint activities because the project has not yet been fully finalized, as phase IV has yet to be carried out. At the moment, during community meetings, some villagers are helping to select the plants that will be the subject of participatory management plans, and two villagers have asked for help in creating a green seal for the forest products they want to develop (soaps, ointments and candles from plants in the Atlantic Forest). However, we note that only a small part of the community engages in future activities or throughout the whole process. This seems reasonable, since people have different interests in all societies and situations.
6. Bring together researchers, community members and the other stakeholders involved through discussions and joint planning. In our experience, the managers of the parks where the *quilombolas* are located played an important role in terms of calling for researchers to address issues related to the community use of natural resources. However, as the project developed, there was practically no interaction between park managers, university researchers, and local communities. The lack of dialogue among the various stakeholders made it difficult to address questions from residents about land use possibilities (e.g., among others, wood extraction and the necessity of management plans for exotic species in the Atlantic Forest), thus generating insecurity.

## Conclusions

Methods for participatory ethnobotanical research focused on conservation are being developed. Information about the limits, difficulties and challenges recorded from all of our experiences in different countries and cultures can make a difference in the construction of future approaches that will be diversely suitable in various contexts. Additionally, our experience shows that it is possible to train community members who want to document their knowledge with or without the participation of universities and other research institutes, and this is of great importance since many issues related to intellectual property have been debated in the world.

## Data Availability

Data on the ethnobotanical uses of plants are presented in this article (Tables 1 and 2).

## Abbreviations

*COTEC (Comissão Técnico-Científica do Instituto Florestal*): Technical and Scientific Committee of the Forestry Institute QC *Quilombo do Cambury* QF *Quilombo da Fazenda* *SISBIO (Sistema de Autorização e Informação em Biodiversidade)* Biodiversity Information and Authorization System *SISGEN* (Sistema Nacional de Gestão do Patrimônio Genético e do Conhecimento Tradicional Associado) National System for Management of Genetic Heritage and Associated Traditional Knowledge
